# Neural subnetwork signatures distinguishing source and item memory retrieval: A meta-analysis of 66 fMRI studies

**DOI:** 10.1162/IMAG.a.124

**Published:** 2025-08-22

**Authors:** Hongkeun Kim

**Affiliations:** Department of Rehabilitation Psychology, Daegu University, Gyeongsan-si, Gyeongsangbuk-do, Republic of Korea

**Keywords:** fMRI, meta-analysis, episodic memory, source memory, default mode network, frontoparietal network

## Abstract

This meta-analysis of 66 functional magnetic resonance imaging (fMRI) studies investigated neural subnetworks underlying source versus item memory retrieval, emphasizing functional distinctions within major intrinsic brain networks. Results revealed clear differences in subnetwork activation patterns between the two retrieval types. Within the Frontoparietal Network, Subnetwork A exhibited stronger activation during source retrieval, highlighting its key role in managing cognitive control processes necessary for complex memory tasks; in contrast, Subnetworks B and C showed minimal or no task-specific engagement. Conversely, within the Default Mode Network, Subnetworks A and B were selectively activated during item memory retrieval, suggesting their contribution to the increased subjective vividness characteristic of simpler recollective experiences, while Subnetwork C remained inactive across both retrieval conditions. Lastly, within the Ventral Attention Network, Subnetwork B—but not Subnetwork A—was more active during source than item memory retrieval, possibly reflecting its specific role in coordinating neural activity under the heightened demands of complex retrieval. Together, these findings advance understanding of subnetwork-specific roles in episodic memory retrieval and highlight the utility of subnetwork-level analyses for uncovering detailed functional specialization within large-scale brain networks.

## 1 Introduction

### 1.1 Study purpose

Functional magnetic resonance imaging (fMRI) studies of episodic memory retrieval typically employ one of two paradigms: item memory or source memory tasks. In item memory tasks, participants study a list of items and are later asked to discriminate previously encountered (“old”) items from novel (“new”) ones. In contrast, source memory tasks require encoding each item along with specific contextual features, such as its spatial location or the type of judgment made during encoding (e.g., size or animacy). At retrieval, participants must both recognize the item and recall its associated context. Although source memory tasks are procedurally and analytically more complex than item memory tasks, they are increasingly used due to their presumed sensitivity to recollection—the vivid re-experiencing of past events accompanied by contextual detail (Yonelinas et al., 2024).

The present study investigates how large-scale intrinsic brain networks are differentially engaged during source versus item memory retrieval, using a comprehensive neuroimaging meta-analysis. An expanding body of evidence indicates that the distributed activity observed during cognitive tasks reflects coordinated engagement of intrinsic brain networks rather than isolated regional responses (Bullmore & Sporns, 2009; Kong et al., 2019; Yeo et al., 2011), highlighting the importance of adopting a network-level perspective. However, prior neuroimaging studies directly comparing source and item memory retrieval (Achim & Lepage, 2005a; Fan et al., 2003; Giovanello et al., 2004; Hayes et al., 2011; Mitchell & Johnson, 2009; Monge et al., 2018; Rugg et al., 2012; Slotnick et al., 2003) have largely focused on individual brain regions, often overlooking broader network dynamics. Moreover, even studies that attempted network-level comparisons frequently relied on qualitative assessments, such as visual inspection, rather than statistically robust analyses.

As neuroimaging studies of source and item memory retrieval have steadily accumulated, it has become increasingly difficult to determine which activation patterns are robust across experiments—let alone to clearly differentiate the neural correlates of the two retrieval types. A key strength of neuroimaging meta-analysis—the primary method employed in this study—is its ability to identify consistent activation patterns across diverse experimental contexts (Fox et al., 2014). Two prior meta-analyses, one focusing on item memory retrieval (Kim, 2013) and the other on source memory retrieval (Kim, 2020), included direct comparisons between the two task types as part of broader analyses. Both reported stronger activation in the Frontoparietal Network (FPN) for source memory retrieval. However, evidence for item-preferential activations was inconclusive: the earlier study found no significant effects, and the latter did not explicitly test this contrast.

Concurrently, functional connectivity and related research has demonstrated that large-scale brain networks, such as the FPN and the Default Mode Network (DMN), comprise multiple functionally distinct subnetworks (for a review, see Eickhoff et al., 2018). For instance, the DMN has been shown to include at least three separable functional subsystems (Andrews-Hanna et al., 2010; Kong et al., 2019; Yeo et al., 2011). Building on these findings, the present study compares source and item memory retrieval at the subnetwork level—an analytical resolution not previously addressed in individual studies or prior meta-analyses. Departing from earlier approaches that relied primarily on visual inspection, this study employs statistically rigorous methods within the Yeo 17-network cortical parcellation framework (Yeo et al., 2011). By integrating fine-grained network analysis with enhanced methodological rigor, this study seeks to elucidate the subnetwork-level functional architecture underlying source and item memory retrieval—and episodic retrieval more broadly.

### 1.2 Frontoparietal network and default mode network

Although the present study systematically examines all major intrinsic brain networks, particular emphasis is placed on the FPN and DMN, given that most significant activations in fMRI studies of episodic memory retrieval fall within these two networks. The following section reviews the presumed functions of the FPN and DMN during retrieval and uses this framework to formulate hypotheses for the upcoming meta-analytic results. While the study aims to elucidate the roles of FPN and DMN subnetworks (which are described in the Methods section), the hypotheses are framed at the level of the parent networks—that is, FPN and DMN—due to the limited current understanding of subnetwork-specific functions. The results of this study are expected to help address this gap in knowledge.

The FPN, widely recognized for its central role in controlled cognitive processing (Dosenbach et al., 2006; Duncan, 2010; Niendam et al., 2012), has been implicated in supporting controlled retrieval operations (St. Jacques et al., 2011; Kim, 2020; Rugg, 2024). Supporting this interpretation, FPN activity increases during memory retrieval under conditions of heightened cognitive demand—particularly when memory confidence is low and strategic control is required (Chua et al., 2009; Fleck et al., 2006; Hayes et al., 2011; Hutchinson et al., 2015; Kim & Cabeza, 2009).

In contrast, the DMN is primarily associated with internally directed processes such as future thinking, self-reflection, mind-wandering, and mentalizing (Addis et al., 2007; D’Argembeau et al., 2008; Mars et al., 2012; Smallwood et al., 2012). These functions have led to the hypothesis that the DMN contributes to the subjective experience of episodic remembering, with its activation reflecting the vividness or clarity of recollected events (Kim, 2020; Rugg & Vilberg, 2013; Simons et al., 2022). Supporting this view, DMN activity increases during episodic retrieval when memories are particularly vivid and confidence in memory judgments is high (Daselaar et al., 2006; Hayes et al., 2011; Hutchinson et al., 2015; Kim & Cabeza, 2009; Moritz et al., 2006).

Building on these conceptualizations of the FPN and DMN, the present study addresses two central questions regarding source and item memory retrieval. First, because source memory tasks involve retrieving contextual details in addition to item recognition, they are inherently more complex and impose greater demands on controlled retrieval processes than item memory tasks. Consistent with this view, prior meta-analyses have reported stronger FPN activation during source compared to item memory retrieval (Kim, 2013, 2020). The current study examines whether this increased engagement is uniformly distributed across all three FPN subnetworks identified in Yeo et al.’s 17-network parcellation, or whether particular subnetworks are disproportionately involved in supporting source memory retrieval.

Second, item memory tasks may evoke a more vivid or coherent recollective experience than source memory tasks, as they often rely on more readily accessible memory traces. Based on this hypothesis, item memory retrieval is expected to elicit stronger engagement of the DMN. However, the only prior meta-analysis to investigate item-preferential effects (Kim, 2013) found no supporting evidence—possibly due to limited statistical power, as it included only five source memory studies. Leveraging a substantially expanded dataset, the present study reexamines this hypothesis and, if supported, evaluates whether enhanced DMN activation during item memory retrieval is broadly distributed across all three DMN subnetworks defined in Yeo et al.’s 17-network parcellation, or whether specific subnetworks are preferentially involved.

## 2 Methods

### 2.1 Data collection process

To identify candidate studies for inclusion in this meta-analysis, an extensive literature search was conducted in the PubMed database in December 2024 using the following keywords: fMRI AND (“source memory” OR “recollection” OR “retrieval success” OR “old/new recognition”) AND retrieval. This primary search was supplemented with a parallel query in the Google Scholar database. The search pool was further expanded through a careful review of references cited in previous meta-analyses on episodic memory retrieval. All studies identified through these methods were subjected to rigorous screening based on predefined inclusion and exclusion criteria, as outlined below.

#### 2.1.1 Foundational criteria

The foundational criteria for study inclusion in this meta-analysis were as follows. First, studies were required to employ fMRI as the primary investigative method. Second, only studies involving healthy participants were included to ensure sample consistency. Third, studies had to report peak activation coordinates in a standardized stereotactic space—specifically, either Talairach or Montreal Neurological Institute (MNI) coordinates.

#### 2.1.2 Selection of item memory studies

Experiments (defined as distinct reported contrasts) included in the item memory analysis were limited to those comparing correct recognition of studied items (hits) with correct rejection of novel items—a standard practice in item memory research. This contrast, however, confounds memory response with item type, because old and new items differ not only in mnemonic status but also in prior exposure. Although a hits-versus-misses contrast avoids this confound by holding item type constant, its infrequent use makes it unsuitable for meta-analysis. For this reason, the present study employed the hits-versus-correct-rejections contrast. Although some source memory studies reported item memory contrasts—typically item-only hits versus correct rejections—these contrasts were excluded to preserve a clear distinction between item and source memory retrieval and to reduce potential confounds.

#### 2.1.3 Selection of source memory studies

The primary focus of source memory studies is on trials in which participants successfully retrieve both item-specific and contextual information—referred to as *source-correct* trials. Accordingly, only experiments that contrasted source-correct trials with correct rejections of unstudied items—a standard comparison in this paradigm—were included in the present analysis. This selection ensured that both the source and item memory analyses employed the same reference condition: correct rejections. Maintaining this consistency was critical, as including studies with differing baselines would have compromised the interpretability of direct comparisons between source and item memory retrieval—the central aim of this study.

#### 2.1.4 Whole-brain analysis

Studies were eligible for inclusion only if they conducted comprehensive whole-brain analyses. Research that exclusively reported results from predefined regions of interest (ROI) or employed small volume corrections (SVC) was excluded. This criterion is critical, as coordinate-based meta-analyses rely on a fundamental assumption: all brain voxels have an equal a priori likelihood of activation. However, in cases where studies reported both ROI/SVC and whole-brain results, ROI/SVC findings were included only if they met or exceeded the significance thresholds used for whole-brain analyses.

### 2.2 Characteristics of the included studies

Brief summaries of the studies included in this meta-analysis are presented below, organized by source memory and item memory groups. Additional methodological and sample details are provided in Table 1.

**Table 1.** Studies included in the present meta-analyses, grouped by source and item memory retrieval.

Study	N	Retrieval task description (per trial)
*A. Source memory retrieval*		
Bergström et al., 2015	18	Judging whether a word was previously spoken by the participant, previously spoken by the experimenter, or is new, or whether a word was previously related to the participant, previously related to Obama, or is new
DeMaster et al., 2013	34	Judging whether a photograph of an object was previously presented on the left side, previously presented on the right side, or is new
Dobbins & Wagner, 2005	14	Deciding which one of three objects was previously studied with a pleasantness judgment, which one of three objects was previously studied with a size judgment, or which one of three objects is new
Donaldson et al., 2009	26	Judging whether a word was previously presented in red, previously presented in green, or is new
Dulas & Duarte, 2012	30	Judging whether a word (or an object) was previously studied with a size judgment, previously studied with an animacy judgment, or is new
Dulas & Duarte, 2014	42	Judging whether an object image was previously presented in the same color, previously presented in a different color, or is new
Duverne et al., 2008	32	Judging whether a picture of an object was previously studied against a left/green background, previously studied against a right/red background, or is new
Hou et al., 2025	23	Judging whether an object was previously studied, and if judged as studied, moving the cursor to the location that had been occupied by the image at study
King & Miller, 2014	20	Judging whether the referent of a word was previously viewed, previously imagined, or the word is new (Perceive condition)
		Judging whether the referent of a word was previously viewed, previously imagined, or the word is new (Imagine condition)
Lewis et al., 2011	22	Judging whether an object image was previously presented with a neural background, previously presented with a negative background, or is new, or whether the background image previously paired with an object image contained people, did not contain people, or the object is new
Lundstrom et al., 2005	16	Judging whether the referent of a word was previously viewed, previously imagined, or the word is new
Lundstrom et al., 2003	21	Judging whether the referent of a word was previously viewed, previously imagined, or the word is new (Imagined condition)
		Judging whether the referent of a word was previously viewed, previously imagined, or the word is new (Viewed condition)
Morcom et al., 2007	16	Judging whether a photograph of an object was previously studied with a size judgment, previously studied with a living/nonliving judgment, or is new (Easy condition; young subjects)
		Judging whether a photograph of an object was previously studied with a size judgment, previously studied with a living/nonliving judgment, or is new (Hard condition; young subjects))
Morcom et al., 2007	16	Judging whether a photograph of an object was previously studied with a size judgment, previously studied with a living/nonliving judgment, or is new (Easy condition; old subjects)
		Judging whether a photograph of an object was previously studied with a size judgment, previously studied with a living/nonliving judgment, or is new (Hard condition; old subjects)
Morcom & Rugg, 2012	18	Judging whether a word was previously studied with a word format, previously studied with a picture format, or is new (Target condition)
		Judging whether a word was previously studied with a word format, previously studied with a picture format, or is new (Nontarget condition)
Ragland et al., 2006	13	Judging whether a word was previously studied with uppercase/lowercase judgment, previously studied with abstract/concrete judgment, or is new
Raposo et al., 2009	16	Judging whether a word was previously presented in List 1, previously presented in List 2, or is new (Distinctive condition)
		Judging whether a word was previously presented in List 1, previously presented in List 2, or is new (Non-distinctive condition)
Smith et al., 2005	18	Judging whether an object image was previously presented on a neutral background, previously presented on a negative background, previously presented on a positive background, or is new
Turner et al., 2008	16	Judging whether a word was previously viewed, previously imagined, or is new, or whether a word was previously presented in List 1, previously presented in List 2, or is new
*B. Item memory retrieval*		
Achim & Lepage, 2005b	18	Judging whether a clipart image was previously studied
Aminoff et al, 2015	95	Judging whether a word was previously studied
		Judging whether a photograph of a face was previously studied
Angel et al., 2016	60	Judging whether a drawing of an object was previously studied, distinguishing between remembering and knowing (Hard condition)
		Judging whether a drawing of an object was previously studied, distinguishing between remembering and knowing (Easy condition)
Chee et al., 2004	16	Judging whether a word was previously studied (Experiment I)
Chee et al., 2004	13	Judging whether a word was previously studied (Experiment II)
Criss et al., 2013	23	Judging whether a word was previously studied, accompanied by a confidence rating
Daselaar et al., 2001	13	Judging whether a word was previously studied
Daselaar et al., 2003	17	Judging whether a word was previously studied (young subjects)
Daselaar et al., 2003	19	Judging whether a word was previously studied (old subjects with normal memory)
Daselaar et al., 2003	21	Judging whether a word was previously studied (old subjects with reduced memory)
de Zubicaray et al., 2005	14	Judging whether a word was previously studied
Delhaye et al., 2023	26	Judging whether a word was previously studied, accompanied by a confidence rating
Elman et al., 2013	19	Judging whether a photograph of a building was previously studied, accompanied by a confidence rating
Evans et al., 2017	54	Judging whether a word was previously studied
Francis et al., 2016	20	Judging whether a photograph of a scene was previously studied
Gaubert et al., 2017	28	Judging whether a word was previously studied
Gilmore et al., 2019	40	Judging whether a drawing of an object was previously studied
Han et al., 2010	19	Judging whether a word was previously studied, accompanied by a confidence rating
Henson et al., 2000	12	Judging whether a word was previously studied, accompanied by a confidence rating
Henson et al., 2005	22	Judging whether a word was previously studied
Herron et al., 2004	12	Judging whether a word was previously studied
Hoppstädter et al., 2015	16	Judging whether a word was previously studied, accompanied by a confidence rating
Hornberger et al., 2006	17	Judging whether a word was previously studied
Iidaka et al., 2006	16	Judging whether a drawing of an object was previously studied
King & Miller, 2017	28	Judging whether a word was previously studied (Perceive, low target probability condition)
		Judging whether a word was previously studied (Imagine, low target probability condition)
		Judging whether a word was previously studied (Perceive, high target probability condition)
Konishi et al., 2000	13	Judging whether a word was previously studied
Korsnes & Magnussen, 2014	16	Judging whether a drawing of an object was previously studied
Leube et al., 2003	12	Judging whether a photograph of a face was previously studied
Leveroni et al., 2000	11	Judging whether a photograph of a face was previously studied
Lundstrom et al., 2003	21	Judging whether a word was previously studied
Maratos et al., 2001	12	Judging whether a word was previously studied
McDermott et al., 2017	21	Judging whether a word was previously studied
O’Connor et al., 2010	19	Judging whether a word was previously studied
Ofen et al., 2012	69	Judging whether a photograph of a scene was previously studied
Quamme et al., 2010	24	Judging whether a word was previously studied
Ragland et al., 2004	15	Judging whether a word was previously studied
Rivas-Fernández et al., 2021	24	Judging whether a word was previously studied
Roberts et al., 2025	20	Judging whether a word was previously studied (Draw condition)
		Judging whether a word was previously studied (List condition)
Rombouts et al., 2001	9	Judging whether a photograph of a scene was previously studied
Sajonz et al., 2010	29	Judging whether a photograph of a scene was previously studied
Shu et al., 2021	29	Judging whether a word was previously studied
Slotnick et al., 2003	8	Judging whether an abstract visual shape was previously studied
Urquhart et al., 2021	21	Judging whether a word was previously studied, accompanied by a confidence rating
van der Veen et al., 2006	24	Judging whether a word was previously studied
von Zerssen et al., 2001	12	Judging whether a word was previously studied, accompanied by a confidence rating
Wang et al., 2024	44	Judging whether a video clip was previously seen, accompanied by a confidence rating
Yoo et al., 2021	22	Judging whether a photograph of a scene was previously studied, accompanied by a confidence rating

The source memory group included 25 experiments drawn from 19 independent studies, encompassing a total of 411 participants. These experiments contributed 292 peak activation foci. Stimulus types varied across experiments: 12 used words, 11 used objects, one used sentences, and one used a combination of words and objects. The nature of the contextual information required for retrieval also differed: six experiments involved recalling the type of task performed during encoding (e.g., size or animacy judgments); six required retrieval of visual context (e.g., red vs. green color); five distinguished between viewed and imagined items; two involved spatial context (e.g., left or right screen position); two required retrieval of list membership (e.g., List A vs. List B); one involved combined retrieval of location and color; and three alternated between two distinct contextual retrieval tasks (e.g., viewed/imagined vs. list membership judgments).

The item memory group included 52 experiments drawn from 47 distinct studies, comprising a total of 1,113 participants and yielding 632 peak activation foci. Stimulus types varied across experiments: 35 used words, six used scenes, five featured objects, three employed faces, two used abstract shapes, and one utilized video clips.

Regarding statistical power, Eickhoff et al. (2016) recommend including at least 20 independent studies in Activation Likelihood Estimation (ALE) meta-analyses, which was employed in the present study, to ensure sufficient power for detecting moderate effects. The item memory group, comprising 47 studies, comfortably exceeds this threshold, providing robust statistical reliability. The source memory group, comprising 19 studies, closely approaches this benchmark, allowing for reasonable confidence in the reliability of its findings.

### 2.3 Conducting the meta-analysis

The meta-analysis employed the ALE method, developed by Eickhoff et al. (2009) and implemented in the GingerALE software (version 3.02; available at http://www.brainmap.org/ale). ALE statistically integrates peak activation coordinates from independent studies to identify brain regions showing a significantly higher-than-chance likelihood of consistent activation across studies. To ensure a common reference space, coordinates originally reported in Talairach space were converted to MNI space using GingerALE’s built-in transformation tool. To maintain data independence, multiple contrasts derived from the same participant group within a single study were merged into a single dataset prior to analysis.

Meta-analyses for the source and item memory groups were conducted separately. Spatial uncertainty around each reported activation peak was modeled using a three-dimensional Gaussian distribution, with the full width at half maximum (FWHM) individually adjusted according to each study’s sample size. The resulting median FWHMs were 9.33 mm for the source memory group and 9.28 mm for the item memory group. ALE values were computed at each voxel by aggregating Gaussian probability distributions derived from peak coordinates within and across studies. Statistical significance was assessed using an analytically derived null distribution, with cluster-level familywise error correction applied at *p* < .05 and a voxel-level cluster-forming threshold set at *p* < .005.

To directly compare the meta-analytic results of source and item memory, a contrast analysis method developed by Eickhoff et al. (2011) was employed. Studies from both groups were pooled and randomly reassigned into two new groups matching the original sample sizes. Voxel-wise ALE scores were computed for each permutation, and the differences between the resulting ALE maps were recorded. This procedure was repeated 10,000 times to generate an empirical null distribution of voxel-wise differences, which was then used to evaluate the statistical significance of observed differences between the original source and item memory maps. Crucially, by permuting data while preserving the original group sizes, this approach accounts for unequal sample sizes and minimizes associated bias.

Due to a current limitation in GingerALE—specifically, the absence of a built-in cluster-level correction for contrast analyses—a voxel-wise threshold of *p* < .05 and a minimum cluster extent of 500 mm³ were applied. This threshold falls within the range of standard practices reported in previous ALE contrast studies (e.g., Bulut, 2023; Kim, 2024a; Molenberghs et al., 2016; Papitto et al., 2020) and helps reduce false positives by conforming to established methodological conventions in the ALE literature.

For visualization, both the thresholded ALE maps and the contrast map were projected onto a three-dimensional, inflated cortical surface using the Population-Average, Landmark- and Surface-based (PALS) atlas developed by Van Essen (2005).

### 2.4 Analyzing associations with intrinsic neural networks

**Fig. 1.**
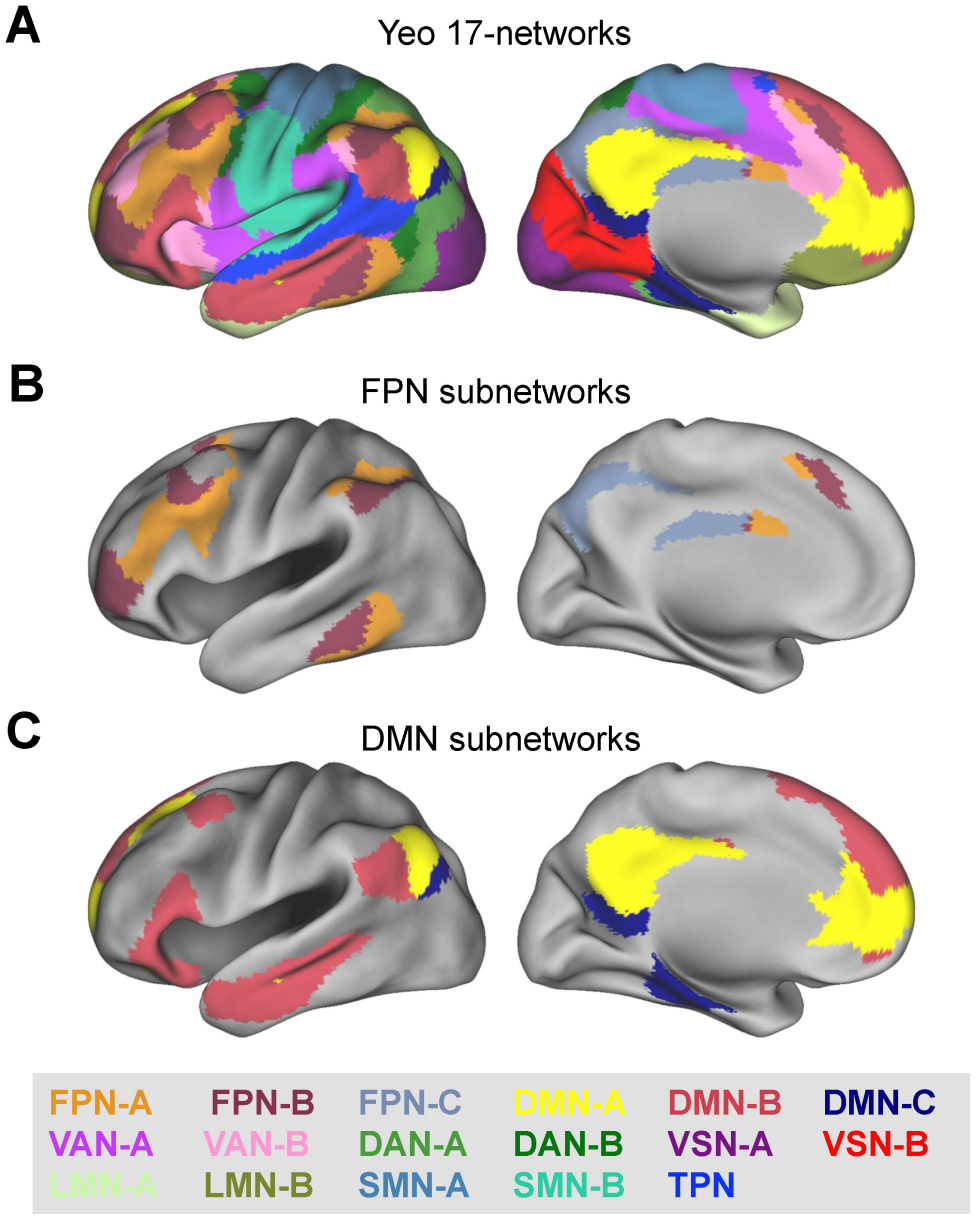
Yeo 17-network parcellation, labeled according to a slightly modified version of the scheme used by Kong et al. (2019). Panel (A) displays all 17 networks. The present study emphasizes subnetworks of the FPN and DMN; for visual clarity, panels (B) and (C) separately present these networks, respectively. The figures are adapted from Yeo et al. (2011) and reproduced with permission from the American Physiological Society. DAN = Dorsal Attention Network; DMN = Default Mode Network; FPN = Frontoparietal Network; LMN = Limbic Network; SMN = Somatomotor Network; TPN = Temporal Parietal Network; VAN = Ventral Attention Network; VSN = Visual Network.

To examine how significant meta-analytic effects are distributed across intrinsic brain networks, the present study utilized the widely adopted Yeo 17-network parcellation framework (Yeo et al., 2011). As the original atlas did not assign formal labels to the networks, a slightly modified version of the labeling scheme adopted by Kong et al. (2019) was used. This scheme subdivides the DMN into three subnetworks (DMN-A, DMN-B, and DMN-C) and the FPN into three subnetworks (FPN-A, FPN-B, and FPN-C), as illustrated in Figure 1.

To quantify the degree of association between identified activation patterns and specific brain networks, the present analysis employed the Network Association Score, introduced by Kim (2024a). This score is calculated as:

$$Network\;Association\;Score=\left(\frac{Overlapping\;Area\;Between\;Effect\;and\;Network\text{​}}{Total\;Network\;Area}\right)\;\times 100$$

This metric provides a standardized measure by adjusting for network size, thereby enabling meaningful comparisons across networks with varying spatial extents. Higher scores indicate a stronger association between the observed effect and the corresponding network.

Statistical significance of the Network Association Score was assessed using a permutation test. First, the number of voxels showing a significant association with a given meta-analytic effect was tallied. These voxels were then randomly reassigned across the 17-network parcellation to simulate the null hypothesis of no preferential network involvement. This randomization procedure was repeated 10,000 times to generate an empirical null distribution of the Network Association Score, against which the observed score was compared. Because the test conditions on the total number of significant voxels, it provides a more sensitive measure than raw voxel-overlap counts: even sparse effects can yield significant associations if the voxels are more tightly clustered within a network than expected by chance. Each comparison was evaluated at *p* < .001. Given 17 networks, a Bonferroni-adjusted α of .05 corresponds to *p* < .00294; the applied threshold of *p* < .001 is therefore more conservative, ensuring robust control of Type I error.

Direct comparisons between two Network Association Scores were performed in targeted analyses—for example, to test whether source memory retrieval was more strongly associated with FPN Subnetwork A than with Subnetwork B. The permutation-based method described above was extended to support these comparisons. For each of the 10,000 permutations, the difference between the two Network Association Scores was computed, generating an empirical null distribution of score differences under the assumption of no true effect. The observed difference was then tested against this distribution, with statistical significance defined as *p* < .001.

## 3 Results

### 3.1 Source memory retrieval

**Fig. 2.**
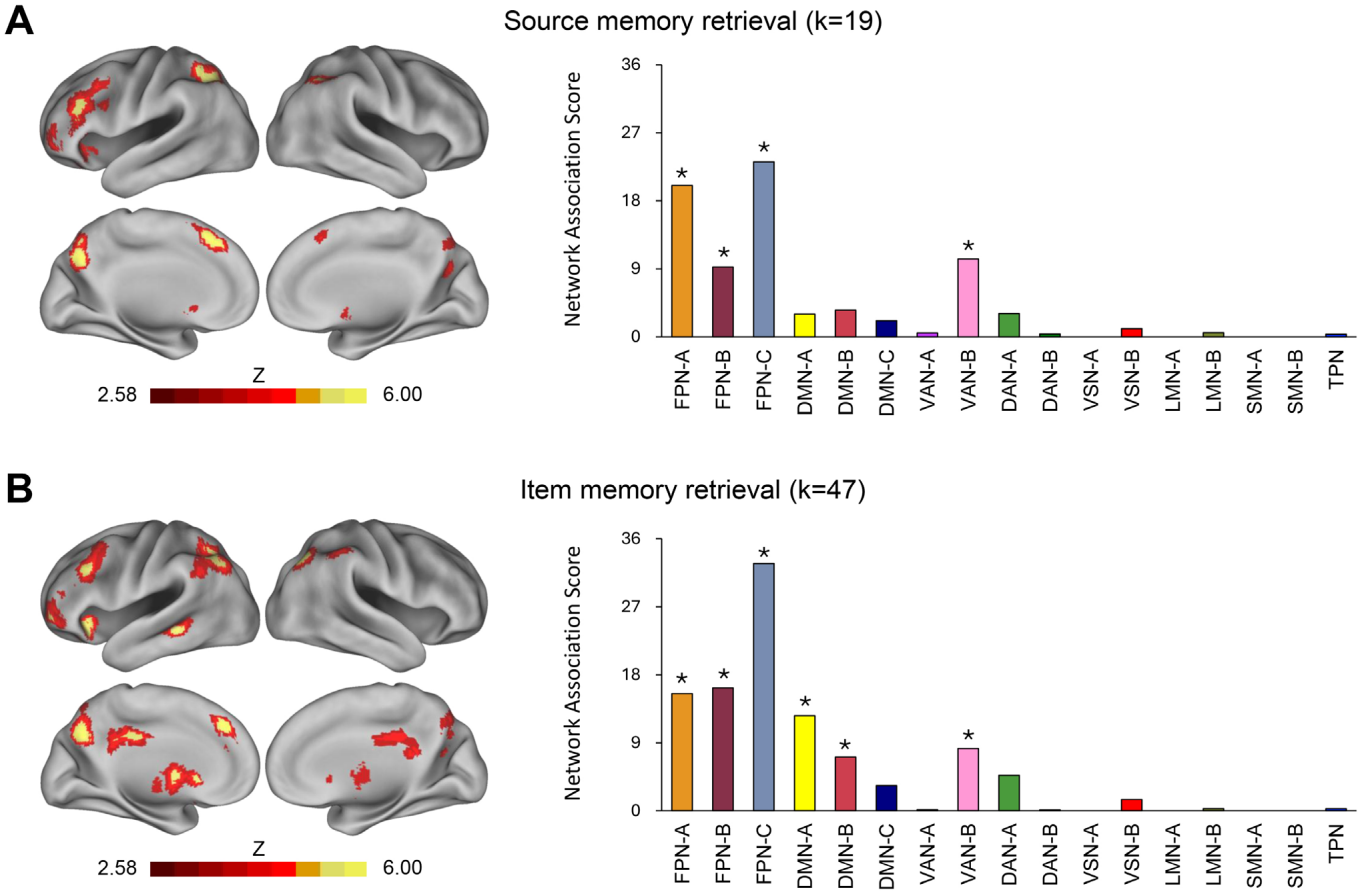
Separate analyses of source and item memory retrieval effects. Panel (A) shows brain regions significantly associated with source memory retrieval, along with their Network Association Scores based on the Yeo 17-network parcellation. Panel (B) presents corresponding results for item memory retrieval. Statistically significant scores (*p* < .001) are indicated by asterisks (*). Bar colors follow the network color scheme introduced in Figure 1 for visual consistency. *k* = number of studies included in the meta-analysis; additional abbreviations are defined in Figure 1 legend.

An ALE meta-analysis was conducted to identify brain regions involved in source memory retrieval, specifically contrasting source-correct trials with correct rejections. Detailed results are presented in Table 2 and visualized in the left panel of Figure 2A. Significant convergence was observed in several prefrontal regions, including the left anterior prefrontal cortex, left lateral prefrontal cortex, left insula, and bilateral superior medial prefrontal cortex. Additional significant convergence was found in the bilateral dorsal parietal cortex, bilateral precuneus, and bilateral caudate nucleus.

**Table 2.** Results of ALE meta-analysis for source memory retrieval, item memory retrieval, and distinctions between them.

			Peak (MNI)				
Lobe	L/R	Region	x	y	z	Z	Volume (mm^3^)
*Source memory retrieval (k = 19)*							
Frontal	L	Anterior/middle/inferior PFC	-48	30	22	6.07	12888
	L	Insula	-36	20	0	4.86	3792
	B	Superior medial PFC	-4	20	48	7.75	5448
Parietal	L	Superior/inferior parietal cortex	-38	-58	50	6.46	7464
	R	Inferior parietal cortex	38	-58	44	5.22	1816
	B	Posterior precuneus	-6	-72	34	6.91	7192
Subcortical	R	Caudate nucleus	12	4	12	4.33	2064
	L	Caudate nucleus	-12	12	-4	5.17	1816
*Item memory retrieval (k = 47)*							
Frontal	L	Anterior/middle PFC	-40	52	0	6.23	6256
	L	Middle PFC	-44	20	34	6.15	5304
	L	Insula	-32	22	-6	8.03	3584
	L	Superior medial PFC	-6	32	38	7.55	3768
Parietal	L	Superior/inferior parietal cortex	-40	-58	46	7.68	10304
	R	Superior/inferior parietal cortex	36	-68	42	6.72	6632
	B	Posterior precuneus	-6	-68	32	6.87	8880
	B	Mid-cingulate cortex	-2	-30	30	5.99	5688
Temporal	L	Middle temporal cortex	-62	-40	-8	5.93	2608
Subcortical	B	Caudate nucleus	-10	12	2	6.88	9448
*Source* *>* *Item*							
Frontal	L	Middle/inferior PFC	-52	31	24	3.89	4896
	R	Posterior middle PFC	29	6	59	2.59	800
	L	Insula	-40	24	6	3.01	864
	B	Superior medial PFC	-11	20	50	3.89	3816
Parietal	L	Superior/inferior parietal cortex	-29	-59	52	2.91	3064
	R	Inferior parietal cortex	38	-54	42	2.62	792
*Item* *>* *Source*							
Temporal	L	Middle temporal cortex	-63	-36	-4	2.14	608

k = number of independent studies analyzed; PFC = prefrontal cortex.

The right panel of Figure 2A shows the Network Association Scores for these regions based on the Yeo 17-network framework. These regions showed significant associations with four networks: all three subnetworks (A, B, and C) of the FPN, and Subnetwork B of the Ventral Attention Network (VAN). Within the FPN, the regions were significantly more strongly associated with Subnetwork A (Score = 20.0) and Subnetwork C (23.2) than with Subnetwork B (9.2; both *p* < .001), yielding a characteristic “V”-shaped profile. Additionally, the association with FPN-C was significantly stronger than that with FPN-A (*p* < .001).

### 3.2 Item memory retrieval

An ALE meta-analysis was performed to identify brain regions associated with item memory retrieval, using a contrast between hits and correct rejections. Full results are reported in Table 2 and illustrated in the left panel of Figure 2B. Significant convergence clusters were found across several prefrontal areas, including the left anterior and lateral prefrontal cortices, left insula, and left superior medial prefrontal cortex. Additional clusters emerged in the left dorsal parietal cortex, bilateral ventral parietal cortex, bilateral posterior precuneus, bilateral mid-cingulate cortex, left middle temporal cortex, and bilateral caudate nucleus.

The right panel of Figure 2B shows the Network Association Scores for these regions. Significant associations were observed with six networks: all three subnetworks of the FPN, Subnetworks A and B of the DMN, and Subnetwork B of the VAN. Both source and item memory retrieval were associated with subnetworks of the FPN and VAN; however, significant associations with DMN subnetworks were unique to item memory retrieval. Within the FPN, Subnetwork C exhibited the strongest association (Score = 32.7), significantly exceeding both Subnetwork A (15.5; *p* < .001) and Subnetwork B (16.3; *p* < .001), whereas the difference between Subnetworks A and B was not significant. This pattern produced a characteristic “L”-shaped profile.

### 3.3 Direct comparison between source and item memory retrieval

**Fig. 3.**
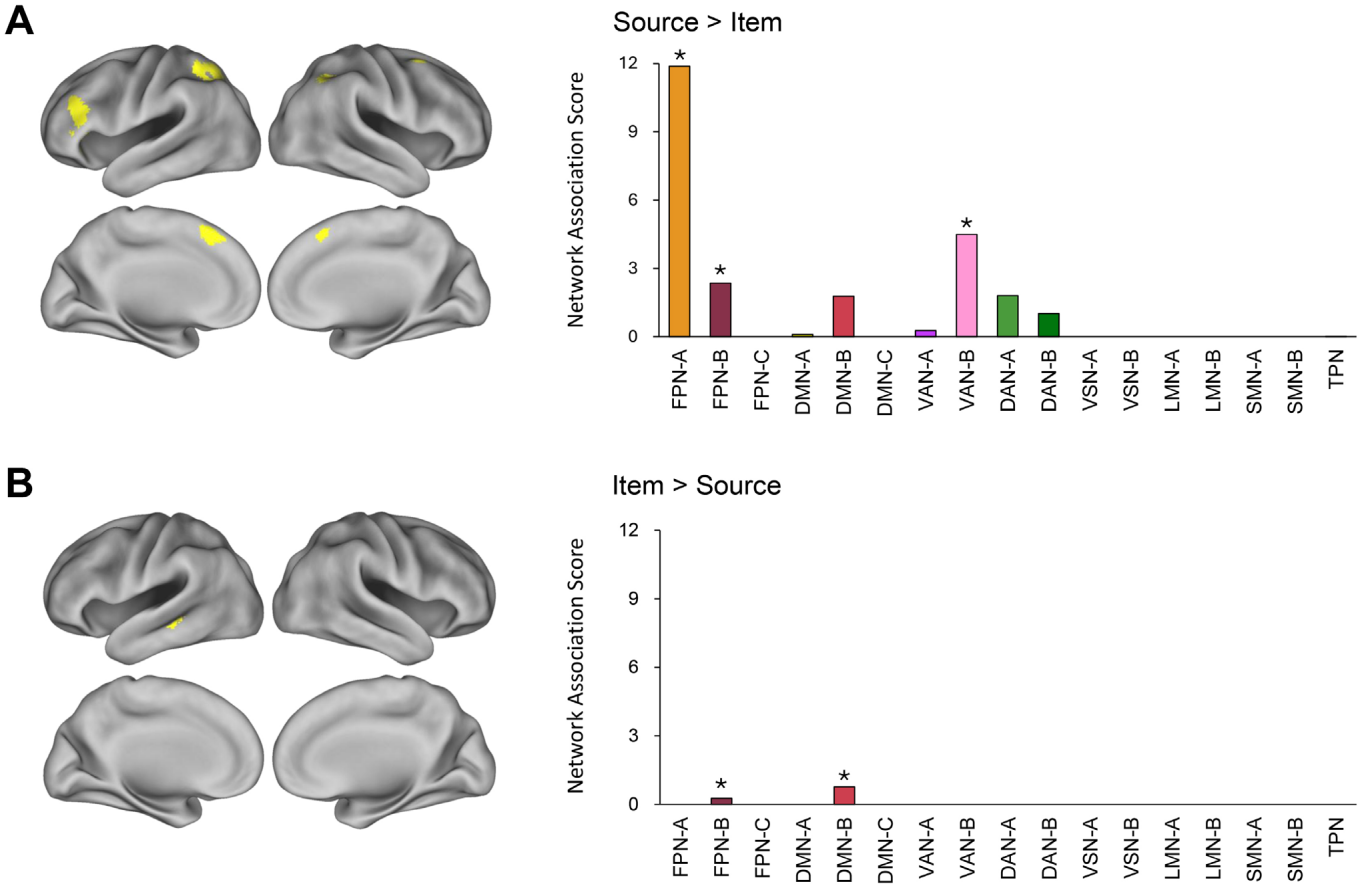
Comparative analyses of source and item memory retrieval effects. Panel (A) highlights brain regions more strongly associated with source memory retrieval than with item memory retrieval, along with their Network Association Scores. Panel (B) shows corresponding results for regions preferentially involved in item memory retrieval relative to source memory retrieval. Statistically significant scores (*p* < .001) are indicated by asterisks (*). Bar colors follow the network color scheme introduced in Figure 1 for visual consistency. For abbreviations, see Figure 1 legend.

The meta-analytic maps for source and item memory retrieval were directly compared to identify brain regions preferentially engaged by each retrieval type. Detailed findings are presented in Table 2 and illustrated in Figure 3.

Source memory retrieval elicited significantly stronger effects than item memory retrieval in several regions, including the left ventrolateral prefrontal cortex, left insula, bilateral superior medial prefrontal cortex, right premotor cortex, and bilateral dorsal parietal cortex (left panel of Fig. 3A). The corresponding Network Association Scores revealed significant associations with three networks: Subnetworks A and B of the FPN, and Subnetwork B of the VAN (right panel of Fig. 3A). Within the FPN, these regions were markedly more strongly associated with Subnetwork A (Score = 11.9) than with Subnetwork B (2.4), a difference that reached robust statistical significance (*p* < .001).

In contrast, item memory retrieval showed significantly stronger effects than source memory retrieval in only one cluster, located in the left middle temporal cortex (left panel of Fig. 3B). The resulting Network Association Scores indicated significant overlap with both Subnetwork B of the DMN and Subnetwork B of the FPN (right panel of Fig. 3B), reflecting the cluster’s anatomical extent: its anterior and middle portions (78.5% of voxels) overlapped with DMN-B, while the posterior portion (21.5%) overlapped with FPN-B.

To resolve this ambiguity, the cluster was re-evaluated using the Yeo 7-network parcellation. Because the parent networks in the 7-network scheme only approximately correspond to the subnetworks in the 17-network scheme—differing slightly in boundary definitions—this additional analysis was warranted. The results revealed that all voxels within the cluster fell exclusively within the DMN, with no overlap with the FPN, clarifying its network affiliation. Accordingly, the apparent association with FPN-B is not considered further.

## 4 Discussion

This study aimed to identify neural subnetwork signatures that distinguish source and item memory retrieval. Distinct patterns were observed within three major networks—the FPN, DMN, and VAN. These findings are discussed in the sections below.

### 4.1 Distinct signatures within the frontoparietal network

Source memory tasks are inherently more complex than item memory tasks, placing greater demands on controlled retrieval processes. This hypothesis predicts stronger involvement of the FPN during source memory retrieval. The meta-analysis supported this prediction, concurrently revealing a pronounced subnetwork dissociation: source memory retrieval elicited markedly greater activation in Subnetwork A compared to item memory retrieval. In contrast, Subnetwork B showed only minimal differences, and Subnetwork C showed no difference. This pattern reflected a distinct “V”-shaped activation profile for source memory, with heightened engagement of Subnetworks A and C relative to B. By contrast, item memory tasks most strongly recruited Subnetwork C, with weaker involvement of Subnetworks A and B, yielding a complementary “L”-shaped profile.

This subnetwork dissociation highlights the pivotal role of Subnetwork A in meeting the elevated cognitive demands of complex retrieval tasks. These demands likely encompass both pre-retrieval operations—such as strategic memory search for difficult-to-access information (Sestieri et al., 2010)—and post-retrieval processes, including memory-related decision making (Henson et al., 2000; Jaeger et al., 2013). Duncan (2010) has identified a domain-general frontoparietal network—the multiple-demand (MD) system—that is consistently engaged across diverse cognitively demanding tasks. The spatial topography of Subnetwork A closely aligns with that of the MD system, suggesting that its strong association with source memory retrieval reflects engagement of this domain-general control system.

The functional dissociation between Subnetworks A and B is particularly notable given that both are large, broadly distributed across frontoparietal regions, and spatially interleaved (Fig. 1B). Dixon et al. (2018) proposed a two-subsystem model of the FPN, in which one subsystem—topographically resembling Subnetwork A—shows stronger connectivity with the Dorsal Attention Network than with the DMN, suggesting a role in externally oriented cognitive control. In contrast, the other subsystem—resembling Subnetwork B—exhibits stronger connectivity with the DMN, implicating it in internally directed processes. Within this framework, Subnetwork A may facilitate the use of external cues to guide memory retrieval, whereas Subnetwork B may contribute to internal operations, such as transforming retrieved content into a decision-relevant mental representation (Wagner et al., 2005). Nonetheless, this interpretation remains speculative, and further research is needed to elucidate the specific contributions of Subnetworks A and B to episodic memory retrieval.

In contrast, Subnetwork C is a relatively small network centered on the precuneus and mid-cingulate cortex. Its spatial profile closely aligns with the “parietal memory network” described by Gilmore et al. (2015), which reliably shows stronger responses to previously encountered stimuli than to novel ones—a phenomenon known as *repetition enhancement*. This effect has been documented across both true and false recognitions (McDermott et al., 2017), as well as during direct and indirect memory tests (Gilmore et al., 2019; Kim, 2018). The present findings extend this pattern, demonstrating that repetition enhancement in this network generalizes across both source and item memory tasks.

Despite its robustness, the functional basis of repetition enhancement in the parietal memory network—corresponding to FPN-C—remains unclear. Competing accounts suggest that it may reflect stimulus familiarity (Gilmore et al., 2015), increased decision conflict for old relative to new items (Aminoff et al., 2015; Jaeger et al., 2013), or the dynamic balancing of attention between external cues and internal memory representations (Lyu et al., 2021; Kim, 2018). Further research is needed to adjudicate among these accounts and clarify the precise role of FPN-C in memory retrieval.

Overall, these findings provide compelling evidence that the FPN—particularly Subnetwork A—is more strongly engaged during source than item memory retrieval, aligning with the interpretation that source retrieval imposes greater demands on controlled retrieval processes. Although this pattern could be alternatively explained by general differences in task difficulty, such an account lacks mechanistic specificity. In contrast, the control-demand hypothesis is grounded in the premise that source retrieval inherently involves greater cognitive complexity than item retrieval, as discussed in the Introduction. Importantly, this account not only aligns with the cognitive demands of the task but also makes anatomically specific predictions—namely, enhanced activation of the FPN. By linking functional demands to identifiable neural substrates, the control-demand framework offers a more theoretically robust and anatomically precise account of the mechanisms supporting source memory retrieval.

### 4.2 Distinct signatures within the default mode network

Item memory tasks may evoke more vivid or coherent subjective recollection experiences than source memory tasks, as they typically rely on more readily accessible memory traces. This hypothesis predicts stronger engagement of the DMN during item memory retrieval. The meta-analysis supported this prediction, concurrently revealing a notable subnetwork-level dissociation: item memory retrieval—but not source memory retrieval—significantly engaged Subnetworks A and B, while Subnetwork C showed no significant involvement in either condition. In direct contrasts, only Subnetwork B demonstrated significantly greater activation during item memory retrieval. Although Subnetwork A did not show a significant difference, this may reflect a relatively weaker effect rather than a true absence of difference, as discussed in detail below.

These findings underscore the critical roles of Subnetworks A and B in shaping the subjective experience of remembering. Subnetwork A is primarily located along midline cortices, whereas Subnetwork B is more broadly distributed across lateral cortical regions (Fig. 1C). These spatial patterns align with the ‘core’ and ‘dorsomedial prefrontal’ subsystems of the DMN, respectively, as defined by Andrews-Hanna et al. (2010). While Subnetwork A’s role in internally directed cognition is well established (Andrews-Hanna et al., 2014; Buckner & Carroll, 2007; Fox et al., 2015; van der Meer et al., 2010), the function of Subnetwork B remains less clear. Its principal regions—including the ventrolateral prefrontal and lateral temporal cortices—overlap with classic language areas, suggesting a role in verbal processing (Andrews-Hanna et al., 2014; Ji et al., 2019). Supporting this, a recent fMRI study (Kim, 2024b) linked Subnetwork B to successful verbal encoding. A plausible retrieval-related function may, therefore, involve verbally or semantically mediated aspects of recollection.

Subnetwork C is a relatively small network encompassing primarily the parahippocampal and retrosplenial cortices. It corresponds to the ‘medial temporal lobe’ subsystem of the DMN identified by Andrews-Hanna et al. (2010) and has been proposed to facilitate the transfer of hippocampal memory signals to other DMN components (Ward et al., 2013). Although its engagement might be expected during retrieval, the current analysis revealed no significant involvement in either item or source memory conditions. A related null finding was the absence of significant hippocampal activation in both contrasts. Hippocampal effects in fMRI are often subtle—characterized by small effect sizes and strong dependence on reference conditions and memory strength (Kim, 2010, 2021; Wixted et al., 2010). Factors that enhance the detectability of hippocampal activity may likewise increase the likelihood of observing engagement of DMN-C, a possibility worth exploring in future research.

Overall, the findings indicate that the DMN—particularly Subnetworks A and B—is more strongly engaged during item than source memory retrieval. However, this item-preferential effect was modest in magnitude compared to the more robust source-preferential engagement observed in the FPN. This asymmetry may reflect a genuine difference in neural recruitment, but it could also arise from methodological factors. One important consideration is potential bias introduced by unequal inclusion criteria: the source memory analyses included only source-correct trials, which likely reflect relatively strongly encoded events, while concurrently excluding item-only-correct trials, which more often reflect more weakly encoded events. By contrast, the item memory analyses included all hit trials without such filtering. Although this approach was intended to permit a direct comparison between successful source and item retrieval, it may have inadvertently introduced selection bias.

Specifically, it may have resulted in comparing strong memories in the source condition with a heterogeneous mix of strong and weak memories in the item condition, potentially underestimating the true extent of DMN engagement for item relative to source memory retrieval. From a recollection–familiarity perspective, this approach may have compared recollection-based recognition in the source memory condition with a mixture of recollection- and familiarity-based recognition in the item condition. Nonetheless, it is important to emphasize that the aim of this study was to contrast successful source and item memory retrieval—not to dissociate memory strength or recollection from familiarity—and the experimental design was structured accordingly. Future research may address these limitations by more directly examining distinctions between objective and subjective recollection.

### 4.3 Distinct signatures within the ventral attention network

Outside the FPN and DMN, only the VAN showed significant involvement in source or item memory retrieval, with a clear subnetwork-specific pattern. Subnetwork B was significantly engaged during both retrieval types, with stronger activation observed for source memory. In contrast, Subnetwork A showed no significant involvement in either condition.

The VAN, originally identified in the context of attentional control (Corbetta & Shulman, 2002), shows substantial anatomical and functional overlap with the salience network described in later research (Seeley et al., 2007). As a result, it is often referred to as the Ventral Attention/Salience Network (Kong et al., 2019; Rieck et al., 2021). This network is well known for detecting salient, behaviorally relevant stimuli and coordinating adaptive neural responses (Goulden et al., 2014; Menon & Uddin, 2010; Seeley et al., 2007). Although firmly established in attentional and salience processing, the VAN has received comparatively little attention in memory research—likely due to its more modest involvement relative to the FPN and DMN. Nonetheless, the present findings suggest that VAN-B may play a meaningful role in episodic memory retrieval.

The functional dissociation between VAN Subnetworks A and B is particularly noteworthy, given their close anatomical interleaving (Fig. 1A). A plausible account of VAN-B’s engagement across both retrieval types is that it reflects the perceived salience of previously encountered stimuli. Participants may implicitly treat old items as behaviorally relevant targets, effectively interpreting the memory task as a variant of target detection (Kim, 2020; O’Connor et al., 2010). The stronger activation of VAN-B during source versus item retrieval may reflect increased demands on neural coordination, in line with the greater cognitive complexity of source memory tasks. These interpretations remain speculative, and further investigation is needed to clarify the role of VAN-B in episodic retrieval.

Recent work by Kwon et al. (2025) demonstrated that the salience and parietal memory networks constitute a unified functional system when network estimation is performed at the individual level using high-resolution functional mapping. Consistent with this finding, the present meta-analysis revealed significant associations of both item and source memory retrieval with VAN-B and FPN-C—networks corresponding to the salience and parietal memory systems, respectively. Their consistent co-engagement may reflect a shared mechanism supporting controlled retrieval and highlights a promising direction for future research into the large-scale network dynamics of memory retrieval.

### 4.4 Conclusions

This study identifies distinct subnetwork-level neural patterns differentiating source and item memory retrieval across three major intrinsic brain networks: the FPN, DMN, and the VAN. Within the FPN, Subnetwork A exhibited substantially greater activation during source memory retrieval compared to item retrieval, underscoring its central role in meeting increased cognitive control demands of complex memory tasks. In contrast, Subnetworks B and C showed minimal or no task-specific differences. Within the DMN, Subnetworks A and B were significantly engaged during item memory retrieval but not during source retrieval, suggesting their critical involvement in the heightened subjective vividness characteristic of simpler retrieval experiences. Subnetwork C remained uninvolved across both retrieval types. Within the VAN, source memory retrieval preferentially activated Subnetwork B—but not Subnetwork A—relative to item retrieval, possibly reflecting its specialized role in coordinating neural responses for complex retrieval. Together, these findings illuminate the distinct subnetwork-level mechanisms that support episodic memory retrieval and underscore the value of fine-grained network analyses for advancing our understanding of the dynamic cognitive architecture underlying memory.

## Data Availability

The data supporting the findings of this study are openly available at https://identifiers.org/neurovault.collection:20036. The code used to reproduce the results is available at https://doi.org/10.13140/RG.2.2.22654.91205.

## Utilization of Generative AI and AI-Assisted Technologies in the Writing Process

While preparing this manuscript, the author used ChatGPT to improve language and readability. After using this tool, the author reviewed and edited the content as needed and takes full responsibility for the content of the publication.
